# Hyperfibrinolysis secondary to acquired factor XIII deficiency A case report

**DOI:** 10.1097/MD.0000000000029446

**Published:** 2022-07-22

**Authors:** Lingsu Gao, Dengju Li, Meiqi Ding

**Affiliations:** a Department of Hematology, The Lu’an Hospital Affiliated to Anhui Medical University, Lu’an People's Hospital, Luan, Anhui 237000, China; b Department of Hematology, Tongji Medical College, Tongji Hospital, Huazhong University of Science and Technology, Wuhan, Hubei, 430000 China.

**Keywords:** aminomethylbenzoic acid, factor XIII deficiency, fresh frozen plasma, hyperfibrinolysis, prothrombin complex

## Abstract

Hyperfibrinolysis induced by factor XIII deficiency (FXIIID) is extremely rare, and patients with no manifestations of active bleeding can easily and frequently be neglected in clinical practice, leading to a missed diagnosis. Herein, we report a rare case of idiopathic FXIIID with secondary hyperfibrinolysis.

**Patient concerns:**

A 69-year-old man presented with ecchymosis of the right arm and chest wall.

**Diagnosis:**

Considering the clinical picture, coagulation function test results, and FXIII activity, the patient was finally diagnosed with hyperfibrinolysis secondary to acquired factor XIII deficiency.

**Interventions:**

The patient was treated with fresh frozen plasma, aminomethylbenzoic acid, a prothrombin complex, etamsylate, dexamethasone, and cryoprecipitate.

**Outcomes:**

The patient improved and was discharged after factor replacement therapy, and no further bleeding was reported 1 month after discharge.

**Conclusion:**

This case report illustrates that the complications of Factor XIII deficiency may include hyperfibrinolysis. Since timely diagnosis of FXIIID is challenging, detailed coagulation factor examinations are needed for definitive diagnosis. It has been suggested that gene testing and antibody testing can help in diagnosis. If ideal treatment is not available, alternative treatment should be provided to reduce bleeding.

## 1. Introduction

Hyperfibrinolysis is mainly found in patients with advanced liver cirrhosis, severe trauma, postpartum hemorrhage, acute promyelocytic leukemia, prostate tumor, or other solid tumors, as well as after plastic surgeries.^[1]^ Hyperfibrinolysis induced by FXIII deficiency (FXIIID) is extremely rare. FXIIID is a rare hemorrhagic syndrome caused by the deficiency/low activity of plasma FXIII, an essential coagulation protein participating in blood coagulation and hemostasis.^[2,3]^ FXIIID is caused by genetic or autoimmune conditions, but the coagulation function of patients with FXIIID mainly shows normal results.^[4]^ Therefore, patients with FXIIID and secondary hyperfibrinolysis but with no manifestations of active bleeding could be easily and frequently neglected in clinical practice, leading to a missed diagnosis. Herein, we report a rare case of idiopathic FXIIID with secondary hyperfibrinolysis.

## 2. Case presentation

A 69-year-old male, Han Chinese, was admitted to the Emergency Department on September 3, 2019, for “ecchymosis in the right arm and chest wall for over 10 days”. The patient was transferred to the hematology department of the hospital on the day of admission. In 2016, the patient had a history of stent implantation for aortic dissection without postoperative oral anticoagulants. On August 31, 2019, the patient was admitted to a local hospital for “ecchymosis in the right arm and chest wall”. Routine blood examination at the local hospital showed a white blood cell (WBC) count of 6.15 × 10^9^/L, hemoglobin (Hb) level of 97 g/L, and platelet count of 73 × 10^9^/L. The coagulation function test showed that the prothrombin time (PT), the activated partial thromboplastin time (APTT) and thefibrinogen (FIB) were 20.4 s (11.5–14.5 s), 45 s(29–42 s), and 0.79 g/L(2.0–4.0 g/L), respectively. The patient was considered to have abnormal coagulation function and was treated with intravenous injection of 0.5 g dicynone (etamsylate) and 30 mg vitamin K1. However, the effects were poor and the manifestations worsened. The patient had a history of hypertension controlled by oral nicardipine sustained-release capsules, betalocol, atorvastatin, and perindopril tert-butylamine tablets. No familial hereditary disease has been reported. He had a history of schistosomiasis. The patient had a regular daily life and was primarily engaged in farming. No smoking or alcohol consumption habits were reported.

Coagulation function was examined in the emergency department of our hospital, which showed that the PT was 17.1 s, APTT was 38.2 s, FIB, 1.16 g/L, and D-dimer (D-D) were 30.9 ng/mL(<0.5 ng/mL). The thromboelastogram (TEG) result was consistent with the symptoms of secondary hyperfibrinolysis, and physical examinations were performed after hospitalization, which showed ecchymosis in the right arm and chest wall. Renal function examination showed that urea was 7.20 mmol/L, creatinine (Cr) was 116 μmol/L, uric acid was 378.0 μmol/L, bicarbonate radical was 26.4 mmol/L, and estimated glomerular filtration rate (eGFR; calculated according to the chronic kidney disease–epidemiology [CKD-EPI] formula) was55.0 mL/min/1.73 m^2^. Liver function examination showed that alanine transaminase was 14 U/L, aspartate transaminase (AST) was 29 U/L, total protein (TP) was 64.4 g/L, albumin was 38.1 g/L, globulin was 26.3 g/L, total bilirubin (TBiL) was 23.6 μmol/L, direct bilirubin (DBiL) was 9.9 μmol/L, and indirect bilirubin (IBiL) was 13.7 μmol/L. The electrolyte levels were within the normal range. Serum protein electrophoresis (SPEP) showed any abnormalities. Routine urine examinations (dry chemistry and urinary sediment) showed a urine specific gravity of 1.0084. Examination of the male tumor markers showed that the squamous cell carcinoma-related antigen (SCCA) was 1.7 ng/mL, and no abnormality was found in the antiphospholipid antibody. Lupus anticoagulant examination revealed no abnormalities. Coagulation function examination showed that PT was 18.2 s, APTT was 56 s, FIB was 1.39 g/L, D-D was 32.71 ng/mL, and fibrin/fibrinogen degradation products (FDP) were 113.1 mg/L(<5 ug/mL). Routine blood examination showed a white blood cell count of 7.99 × 10^9^/L, Hb was 87.0 g/L, and platelet count of 101.0 × 10^9^/L. Routine coagulation factor examinations showed that the endogenous coagulation factors were 107%, 123%, 98%, and 98% for VIII, IX, XI, and XII, respectively. The exogenous coagulation factor VII level was 101%. The coagulation factors for the common coagulation pathway were 56% for II (70%–120%), 32% for V (70%–120%), and 69% for X (70%–120%). Chest and abdominal CT scans showed changes following aortic dissection stent implantation, and the densities of the left subclavian and vertebral arteries were slightly lower. A strip-shaped shadow is observed in the lingular segment of the superior lobe of the left lung. Calculations of the intrahepatic duct or possible calcifications were suggested. Multiple gallbladder calculi, atrophy of the right kidney accompanied by multiple calcifications, and cysts of the right kidney were also found. A linear high-density lesion caused by previous schistosomiasis was found at the rectal wall.

During hospitalization, the coagulation function was tested daily (Table 1). The results showed that the FIB levels were maintained at low levels, while the D-D and FDP levels were elevated, comparable to the levels at admission. Examinations on September 8 and 9 showed FXIII activity of 30.6% and 28%, respectively. According to the symptoms, findings of physical examination, laboratory examination, and imaging examination, abdominal and chest CT scanning, tumor marker examination (which showed no evidence of tumor), and no hemorrhagic disease history of the patient, while FXIII antigen examination showed FXIIID, the patient was diagnosed with FXIIID with secondary hyperfibrinolysis,^[5]^ as well as complications of hypertension, calculus of intrahepatic duct, cholecystolithiasis, right kidney atrophy, and cyst of the right kidney. The FIB levels were decreased, but the liver function was normal, and no previous history of liver disease was reported, which ruled out FIB reduction induced by synthesis disorders. In addition, no FIB reduction was found in 2016 when the patient received surgical treatment, and no familial hereditary disease was reported; thus, hereditary FIB reduction was ruled out. The D-D and FDP levels were increased, while the plasminogen inhibitor levels were reduced (46%; normal range: 80%–120%); thus, the FIB reduction was considered to be caused by secondary hyperfibrinolysis. FXIII antigen was also tested in three children, which showed no abnormalities. The patient's parents had died, and the patient reported no similar diseases in his parents. FXIII gene and antibody were not tested because of insufficient measurement methods.

**Table 1:** Processes of the diagnosis and treatment of the patient.

Location	Time	Coagulation function examination					Factor XIII activity	Blood routine examination	Diagnosis	Treatment	Effectiveness
		PT (s)	APTT (s)	FIB (g/L)	D-D (ng/mL)	FDP (mg/L)					
Local hospital	August 31, 2019	20.4	45	0.79	/	/	/	WBC 6.15'109/L, Hb 97 g/L, platelet 73'109/L	Coagulation disorder was considered	Intravenous dripping of dicynone 0.5 g and vitamin K1 30 mg	Suboptimal, manifestations worsened
Emergency Department of our hospital	September 3, 2019	17.1	38.2	1.16	30.9	/	/	/	/	/	/
Hematology Department of our hospital	September 4, 201*	18.2	35.6	1.39	32.71	113.1	/	WBC 7.99'109/L, Hb 87.0 g/L, platelet 101.0'109/LRenal function: urea 7.20 mmol/L, Cr 116 µmol/L, uric acid 378.0 µmol/L, bicarbonate radical 26.4 mmol/L, eGFR 55.0 mL/min/1.73 m2Liver function: ALT 14 UL, AST 29 U/L, total protein 64.4 g/L, albumin 38.1 g/L, globulin 26.3 g/L, TBiL 23.6 µmol/L, DBiL 9.9 µmol/L, IBiL 13.7 µmol/L. Electrolytes within normal levels.	Acquired XIII factor deficiency, secondary hyperfibrinolysis	Aminomethylbenzoic acid 400 mg + etamsylate 3 g, prothrombin complex 400 U	/
	September 5, 2019	16.6	40.9	1.43	29.93	100.1	/	/		Aminomethylbenzoic acid 400 mg + etamsylate 3 g, prothrombin complex 400 U, fresh frozen plasma 250 mL + dexamethasone 5 mg	Subcutaneous bleeding stopped on this day
	September 6, 2019	16.3	40.6	1.26	28.67	/	/	/		Aminomethylbenzoic acid 400 mg + etamsylate 3 g, prothrombin complex 400 U, fresh frozen plasma 200 mL + dexamethasone 5 mg	/
	September 7, 2019	17.8	38	0.77	23.93	81	/	/		Aminomethylbenzoic acid 400 mg + etamsylate 3 g, prothrombin complex 400 U, fresh frozen plasma 150 mL + dexamethasone 5 mg	/
	September 8, 2019	18.8	42.8	0.8	24.53	/	30.6%	/		Aminomethylbenzoic acid 400 mg + etamsylate 3 g, prothrombin complex 400 U, fresh frozen plasma 500 mL	/
	September 9, 2019	18.1	43.6	0.88	30.48	105.7	28%	/		Aminomethylbenzoic acid 400 mg + etamsylate 3 g, prothrombin complex 400 U, fresh frozen plasma 200 mL, cryoprecipitate 4 U	/
	September 10, 2019	16.7	64	1.4	31.12	/	/	/		/	Subcutaneous hemorrhage generally absorbed

ALT = alanine aminotransferase, APTT = activated partial thromboplastin time, AST = aspartate aminotransferase, Cr = creatinine, DBiL = direct bilirubin, D-D = D-dimer, eGFR = estimated glomerular filtration rate, FDP = fibrinogen degradation products, FIB = fibrinogen, Hb = hemoglobin, IBiL = indirect bilirubin, TBiL = total bilirubin, PT = prothrombin time, WBC = white blood cells.

* Other examinations on September 4, 2019. Serum protein electrophoresis showed no abnormality. Urine routine examinations (dry chemistry + urinary sediment) showed that the urine specific gravity was 1.0084. Examinations of male tumor markers showed that squamous cell carcinoma-related antigen was 1.7 ng/mL. No abnormality was found in the antiphospholipid antibody. Lupus anticoagulant examination also showed no abnormality. Chest and abdominal CT scanning showed changes following aortic dissection stent implantation. The densities of the left subclavian artery and vertebral artery were slightly low. A strip-shaped shadow was found in the lingular segment of the superior lobe of the left lung. Calculus of the intrahepatic duct or possible calcification was suggested. Multiple gallbladder calculi, atrophy of right kidney accompanied by multiple calcifications, and cyst of the right kidney were also found. A Linear high-density lesion was found at the rectum wall, caused by the previous schistosomiasis.

Aminomethylbenzoic acid (400 mg), etamsylate (3 g), and prothrombin complex (400 U) were administered on September 4, and fresh frozen plasma (250 mL) and dexamethasone (5 mg) were added the next day. The subcutaneous bleeding stopped, and no further bleeding manifestations were observed. The same treatment was performed between September 6 and September 9, and September 4. In addition, 150–500 mL of fresh frozen plasma was infused daily according to the supplies from the Blood Transfusion Department. Examination on September 8 still showed that the FIB levels were low, and thus, 4 U of cryoprecipitate was added. Subcutaneous hemorrhage was generally observed on September 10. The coagulation function examination showed that PT was 16.7 s, APTT was 64 , FIB was 1.4 g/L, and D-D was 31.12 ng/mL. The patient was advised to undergo regular coagulation function reexamination. Follow-up by telephone call at 1 month after discharge was performed, and the patient reported no further bleeding.

## 3. Discussion

Herein, we report a rare case of acquired FXIIID with secondary hyperfibrinolysis. Chest and abdominal CT tomography and tumor marker examination showed no signs of tumors. Additionally, the patient had no history of bleeding. FXIII testing revealed FXIII levels of 30%, and the patient was diagnosed with FXIIID and secondary hyperfibrinolysis. Subsequently, replacement therapy was performed. As no concentrated FXIII preparation is available in China yet, replacement therapy by intravenous infusion of prothrombin complex was performed for hemostasis along with fresh frozen plasma (1100 mL in total). Antifibrinolytic treatment with aminomethylbenzoic acid was also administered. The FIB of the patient ranged between 0.77 g/L and 1.43 g/L, but no further hemorrhagic manifestation was found, the subcutaneous hemorrhage absorbed gradually, and the patient improved and was discharged.

FXIIID can be classified into two major types: congenital (hereditary) and acquired. Congenital (hereditary) FXIIID is an autosomal genetic disease, and the incidence is about 1 per 2 million.^[6,7]^ Antibodies against FXIIIa or FXIIIb can be produced in patients with acquired FXIIID due to autoimmune diseases (such as systemic lupus erythematosus [SLE] or rheumatoid arthritis [RA]),^[5]^ solid tumors, lymphoproliferative disease, myeloproliferative neoplasms, or treatment with certain drugs, which could lead to FXIII activity reduction and functional inhibition.^[8]^ Therefore, the main examination for determining the exact nature is FXIII antibody examination.^[8]^ Besides, the half-life of FXIII is about 5–9 days.^[9]^ Therefore, the FXIII levels will not decrease rapidly within a short time after FXIII replacement treatment when no antibody is produced, which can be used as a proxy or supplementary examination.^[8]^ Other available tests include clot solubility testing (but it is no longer recommended) and FXIII functional assays.^[8]^ Exactly distinguishing congenital vs. acquired FXIIID also requires comprehensive examinations including medical history, genetic testing, and mixing and inhibitors studies for immune-mediated acquired FXIIID.^[8]^ Guidelines recommend a first-line FXIII activity assay as a screening test; if FXIII activity is decreased, the subtype should be investigated based on FXIII-A, FXIII-B, and/or FXIII-A_2_B_2_ levels.^[10]^ Due to technical limitations, FXIII antibody examination was not performed for this patient, and thus we could not distinguish whether this patient was with congenital (hereditary) or acquired FXIIID. Nevertheless, the FXIII levels did not decrease after the replacement treatment was performed, the subcutaneous hemorrhage stopped, no further bleeding manifestations were found, and no more bleeding was found within 1 month after discharge, suggesting the possibility of acquired FXIIID.

The coagulation dysfunction in the patient mainly manifested as reduced FIB and increased D-D and FDP levels. Therefore, the patient was diagnosed with hyperfibrinolysis. The plasminogen inhibitor levels in the patient were also reduced, which was in accordance with the diagnosis. Primary hyperfibrinolysis was excluded because D-D and FDP levels are increased at the same time; thromboelastogram showed secondary hyperfibrinolysis, which promptly crosslinks fibrin γ-chains, fibrin α-chains and α 2 -plasmin inhibitor, where FXIII protects newly formed fibrin from the shear stress of circulating blood and from degradation by the powerful fibrinolytic system. We speculated that the reduced FXIII levels of the patient could deprive the protective effects on FIB against fibrinolysis, which then lead to hyperfibrinolysis and FIB reduction. It must be highlighted that the patient underwent stenting and did not receive postoperative anticoagulotherapy, which might have played a role in the onset of coagulation dysfunction. Similarly, Janning et al.^[11]^ reported a patient with acquired factor XIII deficiency who underwent aortic valve reconstruction and replacement of the ascending aorta. However, no definitive causal evidence is available in the literature and requires further study.

Replacement therapy could be provided for patients with FXIIID, for whom concentrated FXIII preparation is preferred. Fogarty et al.^[4]^ reported that a relatively high dose of a concentrated FXIII preparation, approximately 50–150 U/kg, was required for replacement therapy. However, such preparations are not available in China. Therefore, fresh frozen plasma was infused, in which the concentration of FXIII was 3 U/mL.^[12]^ As the patient also had secondary hyperfibrinolysis, aminomethylbenzoic acid was administered. Aminomethylbenzoic acid can directly inhibit the transformation of plasminogen to plasmin and exert its effects by preventing the binding of plasminogen to fibrous proteins. In addition, aminomethylbenzoic acid can also directly inhibit plasminogen activity, but a relatively high dose may be required. Furthermore, aminomethylbenzoic acid could also inhibit the lysis of fibrous proteins, prevent the binding of α_2_-antiplasmin, and inhibit inflammatory reactions.^[13]^ Aminomethylbenzoic acid is effective for the treatment of FXIIID.^[14]^ In this case, bleeding of the right arm and chest wall was gradually absorbed after replacement therapy, and antifibrinolytic therapy was performed. However, FXIII levels did not return to normal, and hyperfibrinolysis did not improve. Therefore, we speculated that higher doses of fresh frozen plasma may be required to increase FXIII levels.

Acquired FXIIID with secondary hyperfibrinolysis are rare. Therefore, the possibility of FXIIID should be considered for patients with idiopathic bleeding. If FXIIID is diagnosed, the causes need to be identified, and specific treatments according to the causes need to be performed. However, owing to the lack of testing technology, the cause of FXIII deficiency cannot be completely eliminated. Replacement therapy can be administered first: infusion of cryoprecipitate or fresh frozen plasma can be considered if concentrated FXIII is not available, and aminomethylbenzoic acid can be used for treatment if the patient is accompanied by hyperfibrinolysis, but both of the required doses of the substitutes are relatively high.

## Acknowledgment

The authors acknowledge the help of Dengju Li, Professor of Hematology at the Tongji Medical College, Tongji Hospital, Huazhong University of Science and Technology, for his guidance on thesis writing.

## Author contributions

Lingsu Gao carried out the study, participated in data collection, and drafted the manuscript. Dengju Li and Meiqi Ding helped draft the manuscript. The authors applied the SDC approach to the sequence of authors. All authors have read and approved the final manuscript.

Supervision: Meiqi Ding

Writing – original draft: Lingsu Gao

Writing – review & editing: Dengju Li

## References

[1] Moore HB Moore EE Gonzalez E. Hyperfibrinolysis, physiologic fibrinolysis, and fibrinolysis shutdown: the spectrum of postinjury fibrinolysis and relevance to antifibrinolytic therapy. J Trauma Acute Care Surg 2014;77:811–7. 25051384 10.1097/TA.0000000000000341 PMC4370273

[2] Schroeder V Kohler HP. Factor XIII: Structure and function. Semin Thromb Hemost 2016;42:422–8. 27019464 10.1055/s-0036-1571341

[3] Muszbek L Penzes K Katona E. Auto- and alloantibodies against factor XIII: laboratory diagnosis and clinical consequences. J Thromb Haemost 2018;16:822–32. 29460500 10.1111/jth.13982

[4] Fogarty H Byrne M O’Connell NM. Acquired factor Xiii deficiency: An uncommon but easily missed cause of severe bleeding. Ir Med J 2018;111:757. 30489053

[5] Ichinose A Kohler HP Philippou H Factor X. Fibrinogen SSCSotI. Recommendation for ISTH/SSC criterion 2015 for autoimmune acquired factor XIII/13 deficiency. Thromb Haemost 2016;116:772–4. 27439329 10.1160/TH16-05-0362

[6] Bouttefroy S Meunier S Milien V. Congenital factor XIII deficiency: comprehensive overview of the FranceCoag cohort. Br J Haematol 2020;188:317–20. 31414482 10.1111/bjh.16133

[7] Lovejoy AE Reynolds TC Visich JE. Safety and pharmacokinetics of recombinant factor XIII-A2 administration in patients with congenital factor XIII deficiency. Blood 2006;108:57–62. 16556896 10.1182/blood-2005-02-0788

[8] Yan MTS Rydz N Goodyear D Sholzberg M. Acquired factor XIII deficiency: a review. Transfus Apher Sci 2018;57:724–30. 30446212 10.1016/j.transci.2018.10.013

[9] Tang N Li D Wang X Yang J. Concurrent hematoma and venous thrombosis in a patient with autoimmune acquired factor XIII deficiency. Int J Lab Hematol 2020;42:e4–6. 31132215 10.1111/ijlh.13053

[10] Kohler HP Ichinose A Seitz R. Diagnosis and classification of factor XIII deficiencies. J Thromb Haemost 2011;9:1404–6. 22946956 10.1111/j.1538-7836.2011.04315.x

[11] Janning M Holstein K Spath B. Relevant bleeding diathesis due to acquired factor XIII deficiency. Hamostaseologie 2013;33(Suppl. 1):S50–4. 24169946

[12] Mitchell JL Lionikiene AS Fraser SR Whyte CS Booth NA Mutch NJ. Functional factor XIII-A is exposed on the stimulated platelet surface. Blood 2014;124:3982–90. 25331118 10.1182/blood-2014-06-583070 PMC4309449

[13] Pabinger I Fries D Schochl H Streif W Toller W. Tranexamic acid for treatment and prophylaxis of bleeding and hyperfibrinolysis. Wien Klin Wochenschr 2017;129:303–16. 28432428 10.1007/s00508-017-1194-y PMC5429347

[14] Ichinose A. Japanese Collaborative Research Group on AH. Autoimmune acquired factor XIII deficiency due to anti-factor XIII/13 antibodies: a summary of 93 patients. Blood Rev 2017;31:37–45. 10.1016/j.blre.2016.08.002 27542511

